# Adjusted Light and Dark Cycles Can Optimize Photosynthetic Efficiency in Algae Growing in Photobioreactors

**DOI:** 10.1371/journal.pone.0038975

**Published:** 2012-06-20

**Authors:** Eleonora Sforza, Diana Simionato, Giorgio Mario Giacometti, Alberto Bertucco, Tomas Morosinotto

**Affiliations:** 1 Dipartimento di Ingegneria Industriale DII, Università di Padova, Padova, Italy; 2 Dipartimento di Biologia, Università di Padova, Padova, Italy; Arizona State University, United States of America

## Abstract

Biofuels from algae are highly interesting as renewable energy sources to replace, at least partially, fossil fuels, but great research efforts are still needed to optimize growth parameters to develop competitive large-scale cultivation systems. One factor with a seminal influence on productivity is light availability. Light energy fully supports algal growth, but it leads to oxidative stress if illumination is in excess. In this work, the influence of light intensity on the growth and lipid productivity of *Nannochloropsis salina* was investigated in a flat-bed photobioreactor designed to minimize cells self-shading. The influence of various light intensities was studied with both continuous illumination and alternation of light and dark cycles at various frequencies, which mimic illumination variations in a photobioreactor due to mixing. Results show that *Nannochloropsis* can efficiently exploit even very intense light, provided that dark cycles occur to allow for re-oxidation of the electron transporters of the photosynthetic apparatus. If alternation of light and dark is not optimal, algae undergo radiation damage and photosynthetic productivity is greatly reduced. Our results demonstrate that, in a photobioreactor for the cultivation of algae, optimizing mixing is essential in order to ensure that the algae exploit light energy efficiently.

## Introduction

Photosynthetic organisms are receiving growing attention, due to their possible exploitation in the production of biofuels for partial replacement of fossil fuels [1]–[5]. One of the possibilities currently implemented is to produce biodiesel from oil-rich seeds, although several problems are still open, such as limited areal productivity of crops and competition with food production for arable land [6]. One interesting alternative is exploiting some species of algae which are capable of accumulating large amounts of lipids and may thus represent suitable feedstock for biodiesel production. These organisms have also been estimated to have potential oil productivity per area which is ten times higher than that of crops, and they are thus a highly promising source for biomass production in a medium-term perspective. However, intensive research efforts are still needed to exploit this potential to the full in large-scale cultivation systems [4], [5], [7].

Algae are a group of organisms with very large biological variability [3]: species of the genus *Nannochloropsis* are particularly interesting in this context, because of their ability to accumulate large amounts of lipids which may reach concentrations of up to 65–70% of total dry weight [8]–[10]. Such massive accumulation of lipids has been shown to be activated in response to stresses such as nitrogen or phosphorus starvation or exposure to excess light [10]–[14].

In order to exploit these organisms for large-scale biofuel production, it is essential to investigate in detail how various parameters influence productivity. Among the several possibilities, light is a major factor, because it provides all the energy necessary to support metabolism but, if present in excess, may lead to the formation of harmful reactive oxygen species (ROS) and oxidative stress [15].

When cells are exposed to illumination, one component of the photosynthetic apparatus, photosystem II (PSII), is continually damaged and must be continually repaired by re-synthesis of damaged components [16], [17]. Photosynthetic organisms exposed to saturating light can also reduce oxidative damage by thermal dissipation of excess energy [15]. Both the repair of damaged photosystems and the dissipation of energy reduce the overall efficiency of light use and should be minimized, if higher productivity is to be achieved.

Algae in photobioreactors are inevitably exposed to variable incident light due to diurnal and seasonal differences in irradiation. *Nannochloropsis* species have been shown to be capable of growing in a large range of illumination intensities, acclimating to changing conditions by optimizing the composition of their photosynthetic machinery to irradiation [8], [18], [19]. Observed responses to different light intensities include modulation of pigment composition and concentrations of enzymes involved in carbon fixation [19], [20].

It should also be noted that algal cultures in photobioreactors have high optical density, which causes highly inhomogeneous light distribution. As a consequence, surface-exposed cells absorb most of the light, leaving only a residual part of the radiation for the cells underneath, which are thus limited in their growth. Instead, external layers are easily exposed to excess light and they must thermally dissipate up to 80% of their photons in order to avoid radiation damage. This greatly reduces their light use efficiency. Following this idea, it has been shown that the overall efficiency of photobioreactors increases when the light path is diminished, reducing the inhomogeneity of light distribution [21]. Unfortunately, very short light paths are difficult to be implemented in large-scale plants, due to practical and economic reasons.

Another factor to be considered is that cells in photobioreactors are rapidly mixed and move abruptly from darkness to full sunlight [22]. Mixing cycles vary greatly according to cultivation system, and mixing rate on a millisecond time-scale can be achieved in closed tubular reactors or optical fiber-based photobioreactors, thanks to turbulent eddies [22]. Conversely, in raceway ponds, laminar flows often affect efficient mixing [23].

Alternation of light/dark periods has been suggested to be beneficial to photosynthetic efficiency [23]–[32]. In some cases, the possibility of achieving light integration has been shown, meaning that fluctuating light can be exploited with the same efficiency as continuous light of equal average intensity [31]. However, the experiments reported in the literature focused on very different ranges of flash frequencies, from 5 Hz [31] to 1 kHz [27], [33], making a complete comparison of results difficult. Also, while some experiments evaluated flash effects only on photosynthetic oxygen evolution [29], [33]–[35], others evaluated longer-term effects such as growth rates [26], [27], [32], which again affected the possibility of merging data.

Experiments with photobioreactors also suggest that mixing rates affect photosynthetic productivity and, in particular, that the latter increases with the frequency of light/dark alternation [25], [36], [37]. However, this conclusion has not always been confirmed, and other reports show that higher mixing rates do not improve photosynthetic efficiency [38], [39], clearly indicating that deeper understanding of the influence of light fluctuations on photosynthetic productivity is needed.

In this work, the influence of illumination conditions on *Nannochloropsis salina* growth and lipid productivity was evaluated in a flat-bed photobioreactor, designed to minimize self-shading. Algae were grown under various continuous irradiances but also with dark/light cycles of different intensities and frequencies, simulating changes in illumination occurring in a photobioreactor as a consequence of mixing. Results showed that, if mixing is appropriately optimized, a photobioreactor can exploit even very intense irradiances with high efficiency. Instead, if the alternation of dark and light is not carefully established, pulsed light can inhibit growth.

## Results

### Growth of *Nannochloropsis salina* in a Flat-bed Panel at Various Illumination Intensities

**Figure 1:**
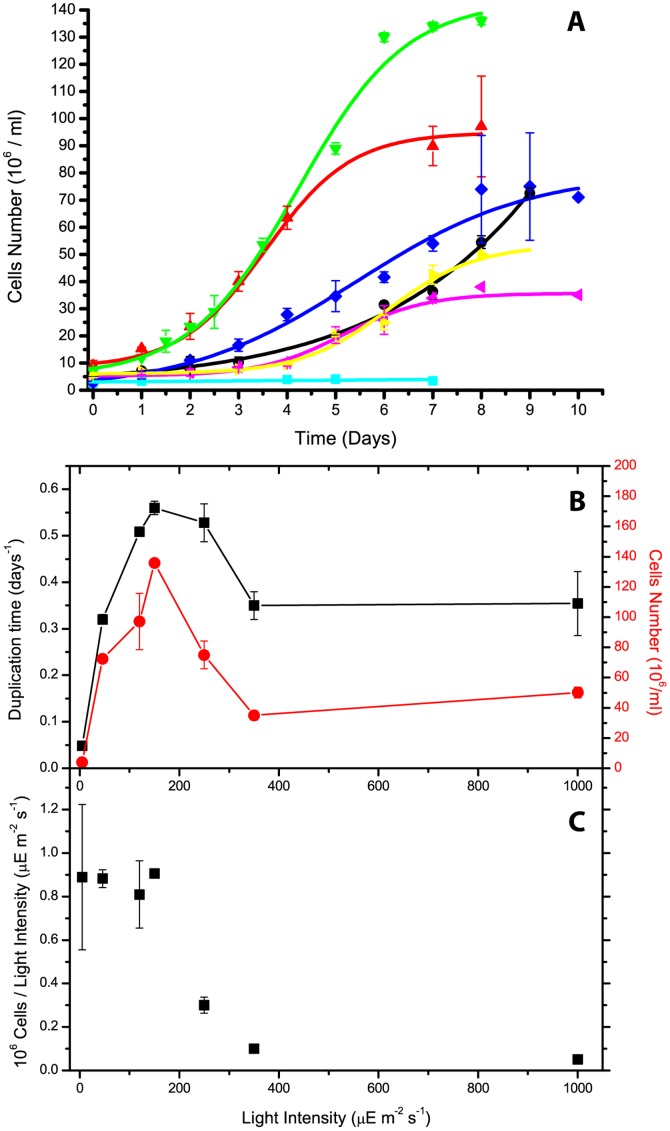
*Nannochloropsis salina* growth under different light intensities in a flat-bed photobioreactor. A) Growth kinetics of algae exposed to differing light intensities from 5 to 1000 µE m^−2^ s^−1^. Data with 5, 50, 120, 150, 250, 350 and 1000 µE m^−2^ s^−1^ shown in light blue, black, red, green, dark blue, pink and yellow, respectively. B) Growth parameters determined from curves in A, specific growth rate (black squares) and cellular concentration after 8 days of growth (red circles). C) Cellular concentrations reported in B normalized to light intensity: this may be used as approximate estimate of biomass production, as no significant deviation of cell size or DW/cell ratio was observed.

The influence of light intensity on *Nannochloropsis salina* growth was assessed in a flat-bed photobioreactor, designed to reduce the influence of self-shading on observed growth rates and productivity (Figure S1). All experiments were performed with low optical density cultures, to further reduce the effect of self-shading on growth kinetics. CO_2_ and nitrogen (as nitrate) were provided in excess, in order to avoid growth limitation due to these nutrients and to reveal illumination effects only. Figure 1 shows that the light intensity reaching the culture greatly influenced both growth rate and final cellular concentration achieved in the stationary phase. Between 5 and 150 µE m^−2^ s^−1^, the growth rate increased with light intensity, peaking at 150 µE m^−2^ s^−1^. Above this limit, any light increase was inhibiting. Under the most intense illumination (350 and 1000 µE m^−2^ s^−1^), growth curves also showed a detectable lag phase, which did not appear in the other conditions (Figure 1A). However, after a few days, *Nannochloropsis* cells resumed growth even in these conditions.

When biomass productivity, expressed as final cellular concentration, is normalized to light intensity, two distinct regions are clearly identifiable. This ratio is constant up to 150 µE m^−2^ s^−1^, indicating that irradiation energy is exploited with comparable efficiency in all cultures within this range. Instead, over the 150 µE m^−2^ s^−1^ limit, the ratio decreases drastically, showing that, although the cells are still capable of showing substantial growth, they use light energy with lower efficiency (Figure 1B).

### Effect of Pulsed Light on Growth of *Nannochloropsis salina*

As already noted, algae in a photobioreactor are subjected to natural variations in illumination but also to dark/light cycles due to mixing. Accordingly, cells rapidly move from regions where they are fully exposed to sunlight, to others where they are substantially in darkness. In order to understand how algae respond to these conditions, *Nannochloropsis salina* cells were grown in square-wave light/dark cycles to simulate mixing. All experiments were performed providing an average total amount of photons always corresponding to 120 µE m^−2^ s^−1^ of continuous light, a value chosen because it represents the highest intensity at which the cell growth is still light-limited. Once the average intensity (I_a_ ) of the light provided was fixed, the influence of other parameters, such as frequency and intensity of light pulses, could be assessed. As shown in Table 1 and schematized in Figure S2, flashes of two different intensities, 350 and 1200 µE m^−2^ s^−1^, were used and, in order to provide the same total amount of energy, light was turned on for one-third and one-tenth of the light cycle, respectively (duty cycles (φ) of 0.33 and 0.1). In both cases, the influence of different frequencies of light changes, corresponding to different durations of light and dark phases, was explored (see Table 1).

**Table 1:** Description of pulsed light conditions employed for *Nannochloropsis salina* growth.

Condition	Light Intensity (I_0_)	Frequency oflight change	Flash time (t_f_)	Dark time (t_d_)	Integrated light intensity (I_a_)	Duty cycle (φ)
**120**	120 µE m^−2^ s^−1^	–	∞	–	120 µE m^−2^ s^−1^	1
**1200-10**	1200 µE m^−2^ s^−1^	10 Hz	10 ms	90 ms	120 µE m^−2^ s^−1^	0.1
**1200-5**	1200 µE m^−2^ s^−1^	5 Hz	20 ms	180 ms	120 µE m^−2^ s^−1^	0.1
**1200-1**	1200 µE m^−2^ s^−1^	1 Hz	100 ms	900 ms	120 µE m^−2^ s^−1^	0.1
**350-10**	350 µE m^−2^ s^−1^	10 Hz	33.33 ms	66.67 ms	120 µE m^−2^ s^−1^	0.33
**350-30**	350 µE m^−2^ s^−1^	30 Hz	11 ms	22 ms	120 µE m^−2^ s^−1^	0.33

Alternating cycles of light and dark were all designed to have same integrated light intensity (I_a_), corresponding to 120 µE m^−2^ s^−1^ of continuous light. Flashes of two intensities were employed, 1200 and 350 µE m^−2^ s^−1^, with duty cycles of 0.1 and 0.33. Light changes made at different frequencies, 10, 5, 1 Hz with 1200 µE m^−2^ s^−1^ and 10, 30 Hz with 350 µE m^−2^ s^−1^. Pulsed light conditions resulted in precise duration of flashes **(t**_f_) and dark **(t**_d_). See also Figure S2.

**Figure 2:**
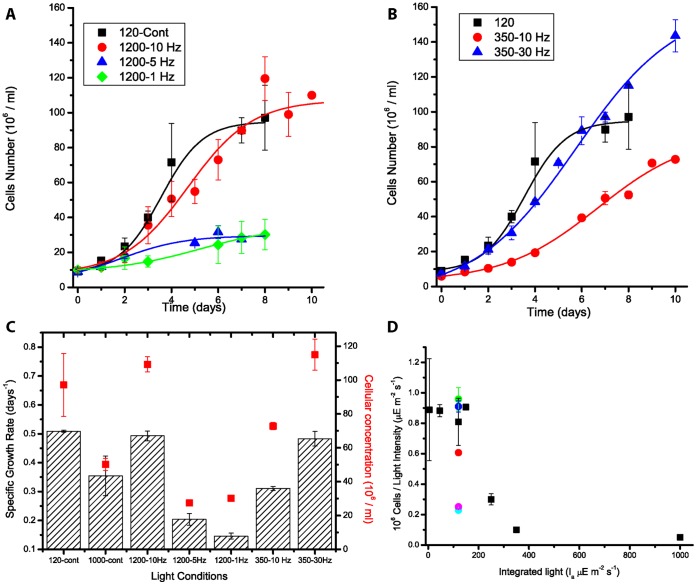
Algal growth kinetics under pulsed light. A–B) *Nannochloropsis* growth curves in pulsed light of differing intensity and frequency, 1200 µE m^−2^ s^−1^ (10, 5, 1 Hz, respectively in red, blue and green, A) and 350 µE m^−2^ s^−1^ (10 and 30 Hz in red and blue, B). Kinetics with 120 µE m^−2^ s^−1^ continuous light reported for comparison (black). C) Growth rate (columns) and cellular concentration after 8 days of growth (red squares) extrapolated from curves in A–B. Values with 120 and 1000 µE m^−2^ s^−1^ constant illumination from Figure 1 reported for comparison. D) Cell concentration in C reported normalized to integrated light intensity. 1200-10, 5, 1 Hz and 350-10, 30 Hz reported in dark blue, pink, light blue, red and green respectively.

With the strongest flashes (1200 µE m^−2^ s^−1^), the choice of the frequency of light pulses showed a huge influence on growth performance. At 10 Hz, the growth rate was the same as that of cells exposed to constant moderate light (120 µE m^−2^ s^−1^, Figure 2A, with nomenclature reported in Table 1). In these conditions, the cells showed complete light integration, meaning that they exploited pulsed light as well as continuous illumination [31]. When light was supplied in pulses at lower frequencies, such as 5 and 1 Hz, growth was greatly inhibited, although the total amount of light and pulse intensity were the same. In the case of 1200-1 and 1200-5 Hz, growth was even slower than under constant, intense light (Figure 2A–C). Biomass productivity, estimated as the number of cells per unit of light intensity (Figure 2D), showed that cells at 1200-1 and 1200-5 Hz exploit light with greatly reduced efficiency, although the total amount of energy provided is low.

Similar experiments were also performed with 350 µE m^−2^ s^−1^ flashes. In one case, i.e., at 350-30 Hz, growth rate and final cell concentration values were again equivalent to continuous 120 µE m^−2^ s^−1^, showing that cells achieved light integration. In another condition, i.e., 350-10 Hz, growth was inhibited, confirming that the frequency of light changes has an enormous effect on biomass productivity.

### Effect of Illumination Conditions on Photosynthetic Apparatus

**Figure 3:**
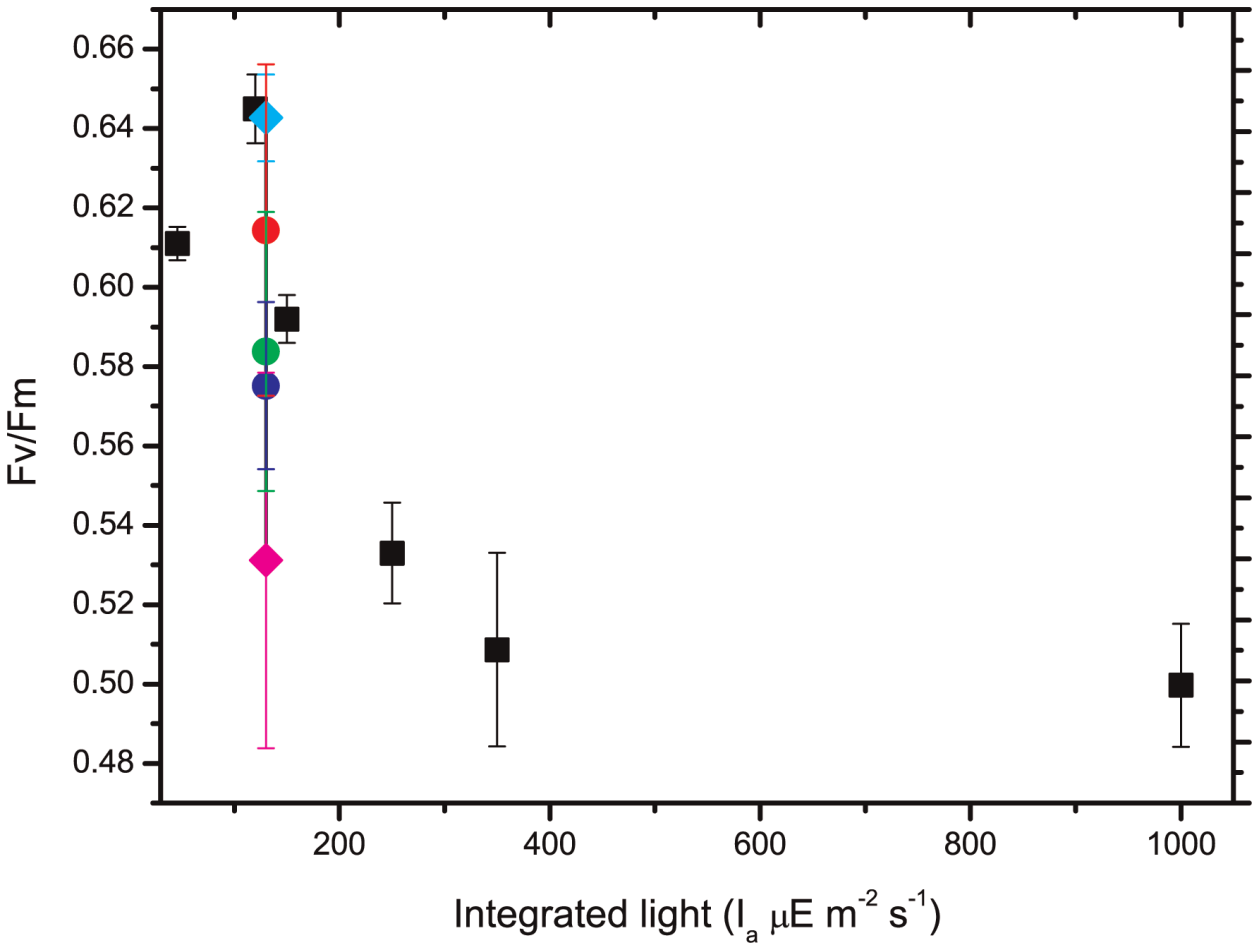
Dependence of photosynthetic efficiency (Fv/Fm) on illumination conditions. Fv/Fm values at end of exponential phase, compared between cells grown under continuous illumination of differing intensity (black squares) and pulsed light of differing intensity and frequency, 1200 µE m^−2^ s^−1^ (10, 5, 1 Hz, red, green and blue circles), 350 µE m^−2^ s^−1^ (10 and 30 Hz, pink and light blue diamonds). Cells at 5 µE m^−2^ s^−1^ were too dilute to provide reliable results.

Fv/Fm is a useful parameter to evaluate photosynthetic efficiency in algae and plants and, in particular, to highlight photoinhibition due to excess illumination [40]. Fv/Fm was monitored in all cultures and cells grown at different levels of continuous light up to 150 µE m^−2^ s^−1^, and all showed similar Fv/Fm values, around 0.62±0.02 (Figure 3). Over this limit, reduced Fv/Fm was correlated with increase of light intensity, indicating that the cells were undergoing photoinhibition.

In pulsed light experiments, Fv/Fm was in the optimal range in both cases when growth was good, 1200 - 10 Hz and 350 - 30 Hz. Instead, in all cases with impaired growth, a reduction in Fv/Fm was also observed, indicating that the cells also underwent photoinhibition, although they were exposed to a low total amount of photons.

**Figure 4:**
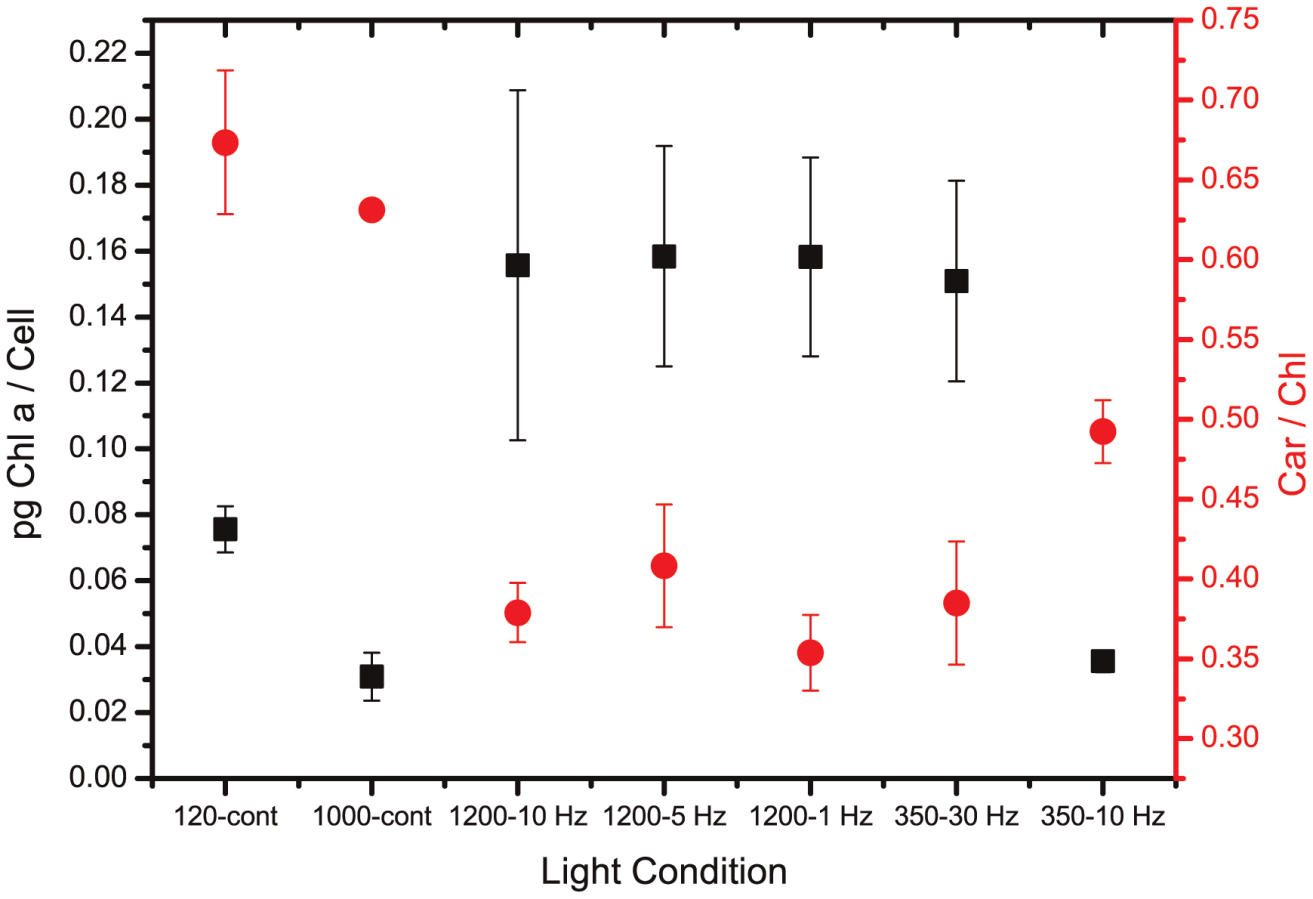
Acclimation response in cells grown in continous vs. pulsed light. Cells grown under differing light intensities, either continuous or pulsed, compared with their Chl content per cell (black) and Chl/Car ratio (red), parameters indicating activation of acclimation response to pulsed light conditions.

*Nannochloropsis*, like many other algae, responds to different light conditions by modulating the composition of its photosynthetic apparatus, a response called acclimation [19], [41]. One regulation commonly observed in photosynthetic organisms exposed to various light intensities is alteration of chlorophyll (Chl) content per cell and the carotenoid (car)/Chl ratio. Under excess illumination, Chl content decreases to reduce light harvesting efficiency, and carotenoids, active in protecting against oxidative stress, are accumulated. As shown in Figure 4, continuous strong light reduces Chl content per cell and increases that of carotenoids.

Instead, clear-cut differences in the light response were observed in cells under pulsed light, which did not accumulate large amounts of carotenoids or even increase their Chl content per cell. The cells thus showed a peculiar response with respect to those exposed to the same total amount of light but provided continuously. This response did not depend particularly on the frequency or duration of light pulses, since similar pigment contents were observed in cells growing well and in others showing light inhibition, with the only exception of the condition 350 - 10 Hz.

### Light-stressed Cells Accumulate Lipids in Excess CO_2_ Conditions Only with Continuous Illumination

Light intensity has been suggested to influence algal lipid synthesis, with strong illumination inducing their accumulation [1], [42]. A transition from control to high light conditions was found to enhance lipid accumulation also in *Nannochloropsis* species [13], [14], [43].

**Figure 5:**
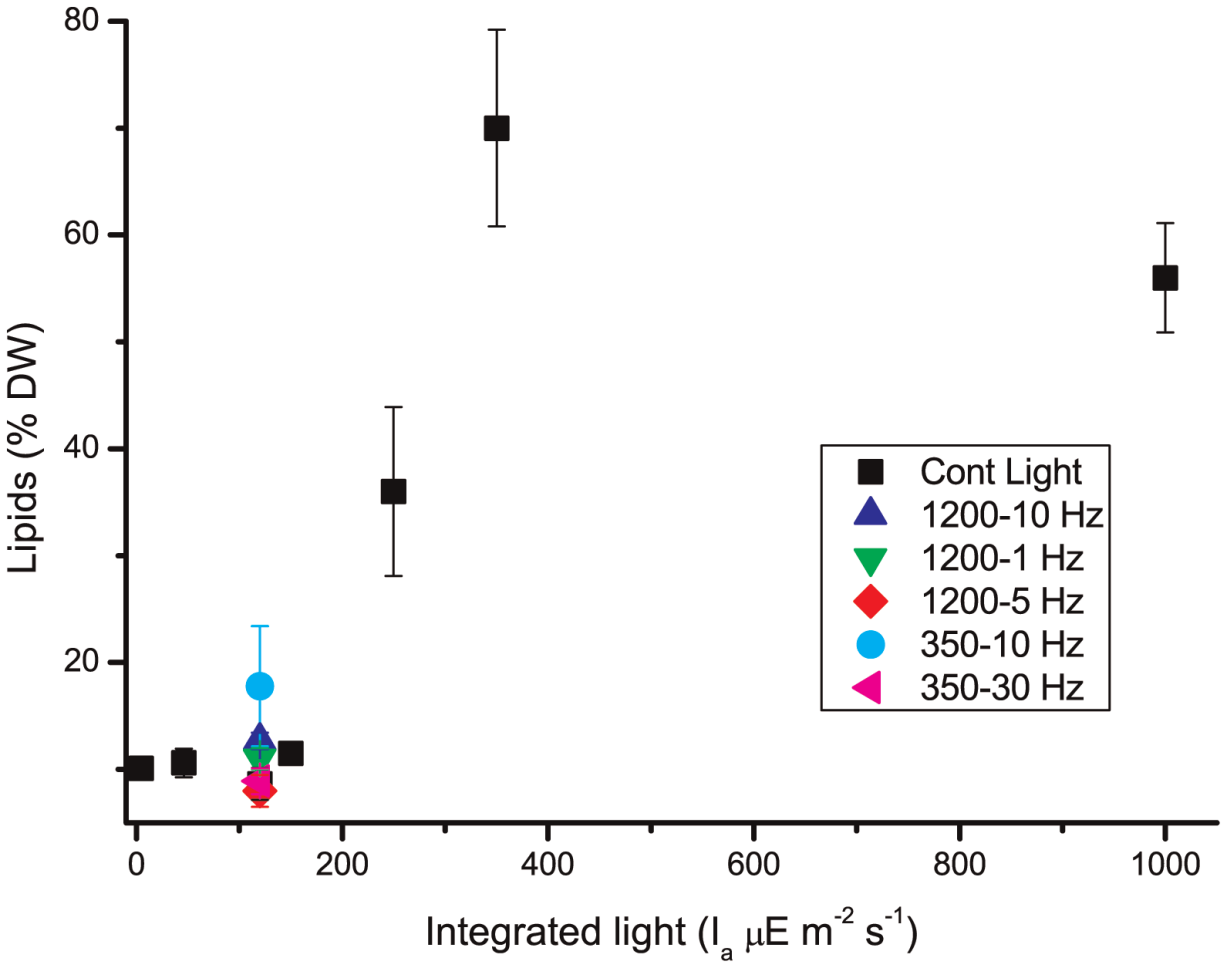
Evaluation of lipid productivity. Dependence of lipid production on illumination intensity. Productivity values with constant light (black squares) compared with the ones with light flashes of 350 and 1200 µE m^−2^ s^−1^ at various frequencies.

To assess if this was also the case in the experimental conditions tested here, lipid productivity was monitored in all cultures (Figure 5). In all limiting light conditions up to 150 µE m^−2^ s^−1^, cells at the end of the exponential growth phase had low lipid contents, around 10% DW, corresponding to the constitutive contents of cellular membranes [44]. At irradiances over 150 µE m^−2^ s^−1^, lipid contents increased, reaching a maximum of 70±9% at 350 µE m^−2^ s^−1^. Even considering that gravimetric evaluation of lipid contents has been suggested to present the risk of overestimation [45], these data support the hypothesis that strong illumination stimulates lipid biosynthesis.

It was interesting to observe that, when cells were grown in pulsed light, no induction of lipid accumulation was observed in any of the conditions tested, whether cells were growing well or not.

## Discussion

### Influence of Light Conditions on Algal Productivity

The effects of light irradiance on *Nannochloropsis* productivity were monitored in a flat-bed reactor designed to minimize cellular self-shading. Since other major factors influencing algal growth (nitrogen and CO_2_) were supplied in excess, this system was considered as optimal to reveal the influence of illumination on algal productivity.

One method for verifying whether this assumption is correct is to compare growth rate and final cellular concentration. The former is measured in the first few days of the curve, whereas biomass concentration depends on when cell duplication stops. In the presence of a significant cells shading, high light cultures should have beneficial effects in the late exponential phase, reaching higher concentrations. Instead, Figure 1B shows that growth rates and final cellular concentrations both show a very similar dependence on illumination intensity, supporting the previous assumption that shading effects are minimized in this system.

Growth dependence on light intensity underwent a first phase during which irradiation was limiting and a second phase in which light had an inhibitory effect (Figure 1B). This was expected, as light may be limiting for growth but, if in excess, leads to oxidative stress [15]. It is worth noting that, at the highest intensity tested (1000 µE m^−2^ s^−1^), cultures showed growth similar to that at 350 µE m^−2^ s^−1^, indicating that cells can protect themselves from such strong light excess and yet maintain significant biomass accumulation.

In order to demonstrate the influence of illumination on growth, it was interesting to analyze the final cell concentration values, normalized to illumination intensity (Figure 1C). Since no significant deviations in cell size or weight were observed, this ratio represents an estimate of culture biomass productivity. In this case, in which light is limiting for growth, cells clearly exploit radiation energy with very similar efficiency, probably to the maximum in these conditions. Instead, after the 150 µE m^−2^ s^−1^ limit, productivity falls drastically. The case of 250 µE m^−2^ s^−1^ is interesting to be mentioned, since cells still showed a good growth rate, close to the values found at 120 µE m^−2^ s^−1^. However, since the light energy provided was more than double, light use efficiency fell by more than 50%. A real large-scale photobioreactor may be imagined as being composed by a overlap of several of these layers: this result indicates when most external cells are exposed to strong illumination, although they are able to cope with the resulting stress, they use available energy with lower efficiency. This drop in productivity of the more external layers, which are also those absorbing most of the light energy, is definitely detrimental for the overall photobioreactor performance.

One strategy against this limitation is suggested by experiments with pulsed light, which showed that even strong light, beyond the saturation point of photosynthesis, can be efficiently exploited for growth, as demonstrated by the fact that cells grew equally well at 120 µE m^−2^ s^−1^ and 1200-10 Hz. Note that, at 120 µE m^−2^ s^−1^, light is still limiting for growth, as shown in Figure 1B–C, implying that, with this optimal alternation of light and dark, cells can use all the energy of pulsed light with the same efficiency they achieve under continuous low illumination [25]–[27], [31]. This is clearly shown by noting that the number of cells per unit of light is the same at 1200-10, 350-30 Hz and all constant low illumination (Figure 2D). This high efficiency is possible because photochemical intermediates produced in a short flash of intense light can be processed further by enzymatic reactions during the following dark period, so that cells perform time integration of the light energy received.

However, similar experiments, at different frequencies, also showed that alternating dark and light may be detrimental, as demonstrated by the growth inhibition observed in the 1200-1 and −5 Hz curves. These cells showed a decrease in Fv/Fm values similar to that observed under constant strong light, indicating that the inhibition of growth was due to photoinhibition. Even more importantly, when the number of cells per unit of light was considered, cells growing at 1200-1 and −5 Hz used light at very low efficiency. For instance, in the 1200-1 Hz experiment, biomass productivity was as low as in 1000 µE m^−2^ s^−1^ continuous light cultures, although the total energy provided was 8.3 times lower. Apparently, in these cases, cells perform worse that those exposed to continuous light, showing that alternating light and dark can also reduce growth efficiency.

In experiments with alternate light, it should be noted that similar results were obtained with light flashes of 1200 and 350 µE m^−2^ s^−1^, indicating that the intensity of the light pulses does not have much influence (Figure 2C). The data also show that frequency *per se* is not the major parameter determining light influence. On the contrary, the conditions with the highest growth (1200 µE m^−2^ s^−1^ - 10 Hz and 350 µE m^−2^ s^−1^- 30 Hz) have in common the same length of the illumination phase, which thus appears to have the largest influence on biomass productivity among the parameters considered here.

The optimal duration of light pulses was found to be around 10 ms, which is consistent with the suggested PSII turnover rate in whole cells [4], [46]. Accordingly, after photon absorption by the photosystem, 1–15 ms are needed to reset the system, before it is ready to receive another photon [22]. If the illumination is this short, most photons are exploited for photosynthesis and do not lead to the formation of ROS which then causes photoinhibition. These results indicate that even strong light does not cause damage if it only lasts a short time. Conversely, longer exposure allows the generation of ROS and damage and, in this case, the abrupt changes in illumination undergone by the cells are as harmful to the photosynthetic apparatus as constant high light.

A further observation is that a peculiar acclimation response is activated under pulsed light conditions. This response does not depend to any great extent on the frequency or duration of light pulses, since similar pigment contents were observed in cells growing well and in others (1200- 1 and 1200-5 Hz), and also in ones showing light inhibition, with the only exception of the 350-10 Hz level. These results indicate a particular type of acclimation response in pulsed light conditions, which does not depend on the stress perceived by the cells, nor on the total amount of light absorbed. However, this deserves further investigation.

### Light Effect on Lipid Production

In the perspective of exploiting algae as feedstock for biodiesel production, biomass growth must be considered together with lipid productivity. The relationship between light intensity and productivity is complex, and many parameters influence the ability of algae to accumulate lipids. The experiments with continuous illumination presented here showed that, under strong continuous light, lipid accumulation is stimulated, as also previously reported [13], [14], [43]. It is noteworthy that such large lipid production was observed in a medium where nitrogen was provided in excess and was not induced by lack of this nutrient. As shown in Figure S3, lipid accumulation starts in the early phases of culture, when nitrogen cannot be depleted, even if a far higher cell consumption is assumed. This result confirms that excess light can induce lipid accumulation without direct or indirect nitrogen depletion, showing that other signals can induce high lipid accumulation in *N. salina*.

In this context, the seminal influence of carbon dioxide availability should be stressed. Previous results on *Nannochloropsis* cells exposed to various radiation intensities with atmospheric CO_2_ showed light stress without any detectable induction of lipids [19]. On the contrary, in the experiments presented here, where carbon dioxide was externally provided, light excess induced lipid accumulation. These results clearly indicate that light stress alone does not induce lipid synthesis, which is instead the result of more complex regulation. With high light and limiting CO_2_, the efficiency of the Calvin-Benson cycle is probably limiting, with the consequent accumulation of molecules upstream of carbon dioxide fixation. Instead, with excess CO_2_ and strong illumination, molecules downstream of carbon dioxide fixation are more likely to accumulate, eventually triggering triacylglycerol biosynthesis.

This hypothesis is consistent with data obtained with cells under pulsed light. For example, cells exposed to 1200-1 Hz showed light stress and inhibited growth which, however, did not result in any significant lipid accumulation, confirming that light stress alone does not induce lipid biosynthesis, but that its influence is integrated with other metabolic signals.

## Materials and Methods

### Culture Conditions

*Nannochloropsis salina,* from SAG, was always grown in sterile filtered F/2 medium [47], with 22 g/l sea salts from SIGMA, 40 mM TRIS HCl pH 8, SIGMA Guillard’s (f/2) marine water enrichment solution 1×, modified by the addition of a non-limiting nitrogen concentration (NaNO_3_ 1.5 g/L). Cultures were maintained and propagated in the same medium, with the addition of 10 g/l of Plant Agar (Duchefa Biochemie). Growth experiments were performed in a flat-bed apparatus 0.8 cm deep (Figure S1). Pre-cultures were grown at 100 µE m^−2^ s^−1^ in the exponential phase, which was diluted to an Optical Density (OD) of 0.45 at 750 nm. The final volume of the photobioreactor was 150 ml. Constant illumination between 5 and 1200 µE m^−2^ s^−1^ was provided with a LED Light Source SL 3500 (Photon Systems Instruments). The light source was also programmed to generate square-wave dark/light cycles at the desired intensities and frequencies (Figure 2). Parameters describing flashes were flash time (t_f_), dark time (t_d_), duty cycle (φ, corresponding to t_f_/(t_f_ + t_d_ ) [26], [32]), flash light intensity (I_0_) and integrated light intensity (I_a_). Temperature was kept at 23±1°C in a growth chamber. CO_2_ in excess (mixed at 5% v/v with air) was supplied by bubbling, which also mixed cells. The medium was buffered with 40 mM TRIS HCl, pH 8, to avoid alterations due to excess supply of CO_2_. Algal growth was measured by daily changes in optical density OD_750_ (Lambda Bio 40 UV/VIS Spectrometer, Perkin Elmer) and cell numbers were monitored in a Bürker Counting Chamber (HBG, Germany). The specific growth rate was calculated by the slope of the logarithmic phase for number of cells. At least four replicates of all curves/experiments were performed.

*In vivo monitoring of photosynthetic parameters.* Chlorophyll fluorescence was determined *in vivo* at the end of exponential phase of growth using a Dual PAM 100 from WALZ. Fv/Fm parameter was calculated as (Fm-Fo)/Fo [48] after 20 minutes of dark adaptation.

### Pigment Extraction and Analysis

Chlorophyll a and total Car were extracted, at the end of exponential phase of growth, from centrifuged cells of *Nannochloropsis* with 100% N,N’-dimethylformamide for at least 48 hours at 4°C in dark conditions, as in [49]. Pigment concentrations were determined spectrophotometrically with specific extinction coefficients [50], [51].

### Lipid Analysis

Lipid contents was routinely monitored by measuring fluorescence of Nile Red stained cells, after verification of the linear correlation between the fluorescence signal and the total amount of lipids. 2*10^6^ cells were re-suspended in 1.9 ml of de-ionized sterile water with 2.5 µg/mL NR and incubated for 10 minutes at 37°C [52]. Fluorescence was measured on a spectrofluorometer (OLIS DM45), with excitation wavelength at 488 nm and emission at 580±5 nm [53]. Signals from algal autofluorescence and Nile Red alone were subtracted. For verification of fluorescence signal linearity, total lipids were extracted from dried cells with ethanol-hexane (2.5∶1 vol/vol) as solvent in a Soxhlet apparatus for 10 h [54], as reported in the Figure S4. The lipid mass was measured gravimetrically after solvent removal in a rotary evaporator.

## Supporting Information

Figure S1 **Scheme of the Flat Bed Photobioreactor.** The flat-plate photobioreactors were built with transparent materials (polycarbonate) for maximum utilization of light energy. The working volume is 150 ml and the culture is mixed by an air-CO_2_ flow from a sparger placed in the bottom of the panel. The amount of CO_2_ in air is regulated by two flow meters. The gas flow supplies a non-limiting CO_2_ content to the culture. The gas flow for each reactor is regulated using suitable valves.
(TIF)

Figure S2 **Pulsed light conditions utilized for** ***Nannochloropsis salina*** **growth.** Alternated cycles of light and dark were designed to have all the same integrated light intensity (I_a_), corresponding to 120 µE m^−2^ s^−1^ of continuous light. Flashes of two different intensities were employed, 350 and 1200 µE m^−2^ s^−1^, with a duty cycle of respectively 0.33 and 0.1. Light changes were performed with different frequencies, respectively 10 and 33 Hz with 350 µE m^−2^ s^−1^ and 10, 5 and 1 with 1200 µE m^−2^ s^−1^. These pulsed light conditions resulted in precise durations of flashes **(t**_f_) and dark **(t**_d_), as reported in Table 1.
(TIF)

Figure S3 **Timeline of lipids accumulation in** ***Nannochloropsis*** **cells exposed to 350 (black) and 150 (red) µE** **m** ^−**2**^ **s** ^−**1**^ **.** Lipid content was evaluated each day using Nile Red staining correlated to total lipid concentration quantified gravimetrically (see Figure S4).
(TIF)

Figure S4 **Correlation of lipid accumulation in** ***Nannochloropsis*** **evaluated by Nile Red staining and gravimetric analysis.**
(TIF)
