# Supplementary figures and images for: Adjusted Light and Dark Cycles Can Optimize Photosynthetic Efficiency in Algae Growing in Photobioreactors
Source: PLoS One. 2012 Jun 20;7(6):e38975. doi: 10.1371/journal.pone.0038975 (PMC3380057; doi:10.1371/journal.pone.0038975)

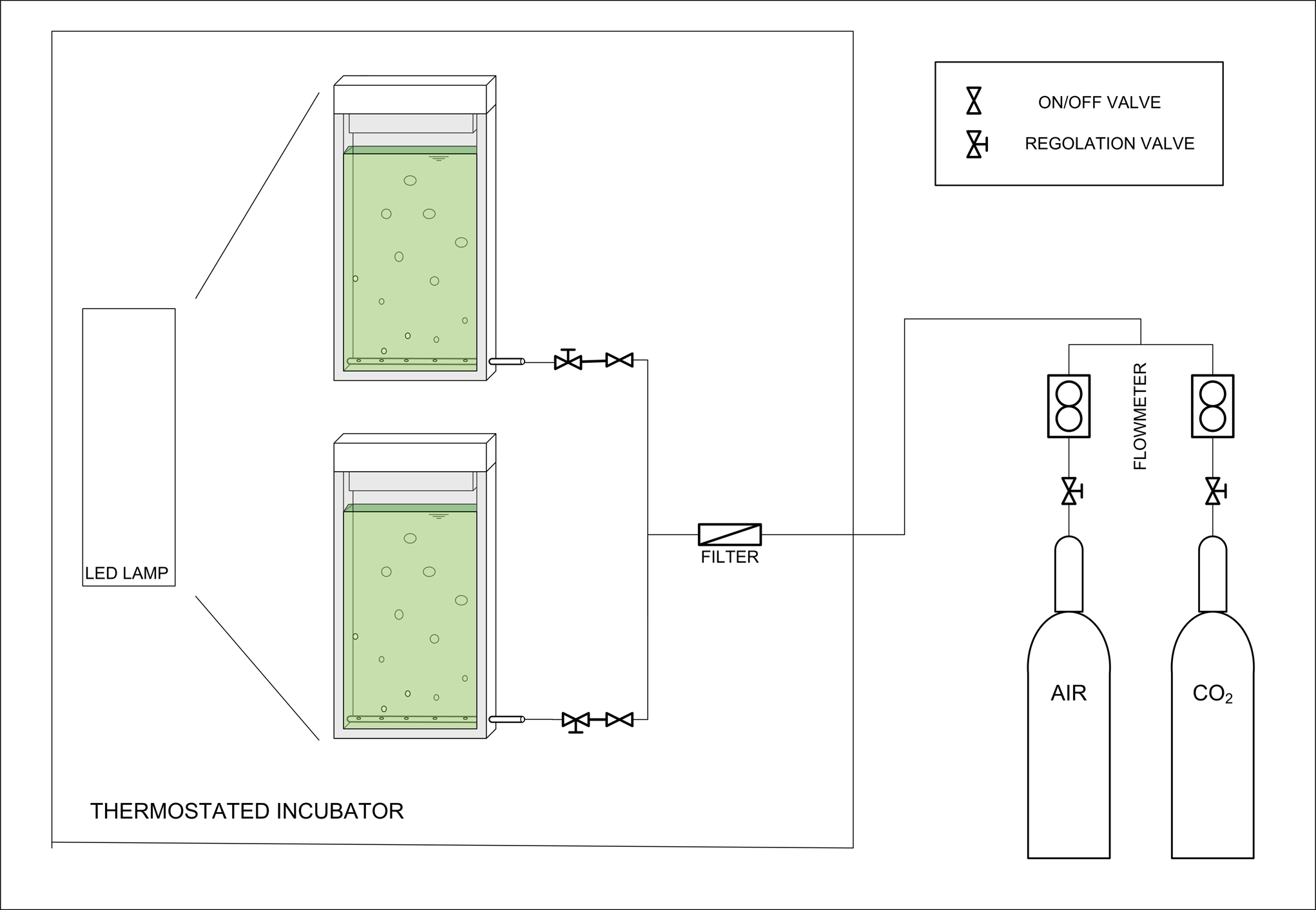

Supplement: Figure S1 — Scheme of the Flat Bed Photobioreactor. The flat-plate photobioreactors were built with transparent materials (polycarbonate) for maximum utilization of light energy. The working volume is 150 ml and the culture is mixed by an air-CO2 flow from a sparger placed in the bottom of the panel. The amount of CO2 in air is regulated by two flow meters. The gas flow supplies a non-limiting CO2 content to the culture. The gas flow for each reactor is regulated using suitable valves. (TIF) [file pone.0038975.s001.tif]

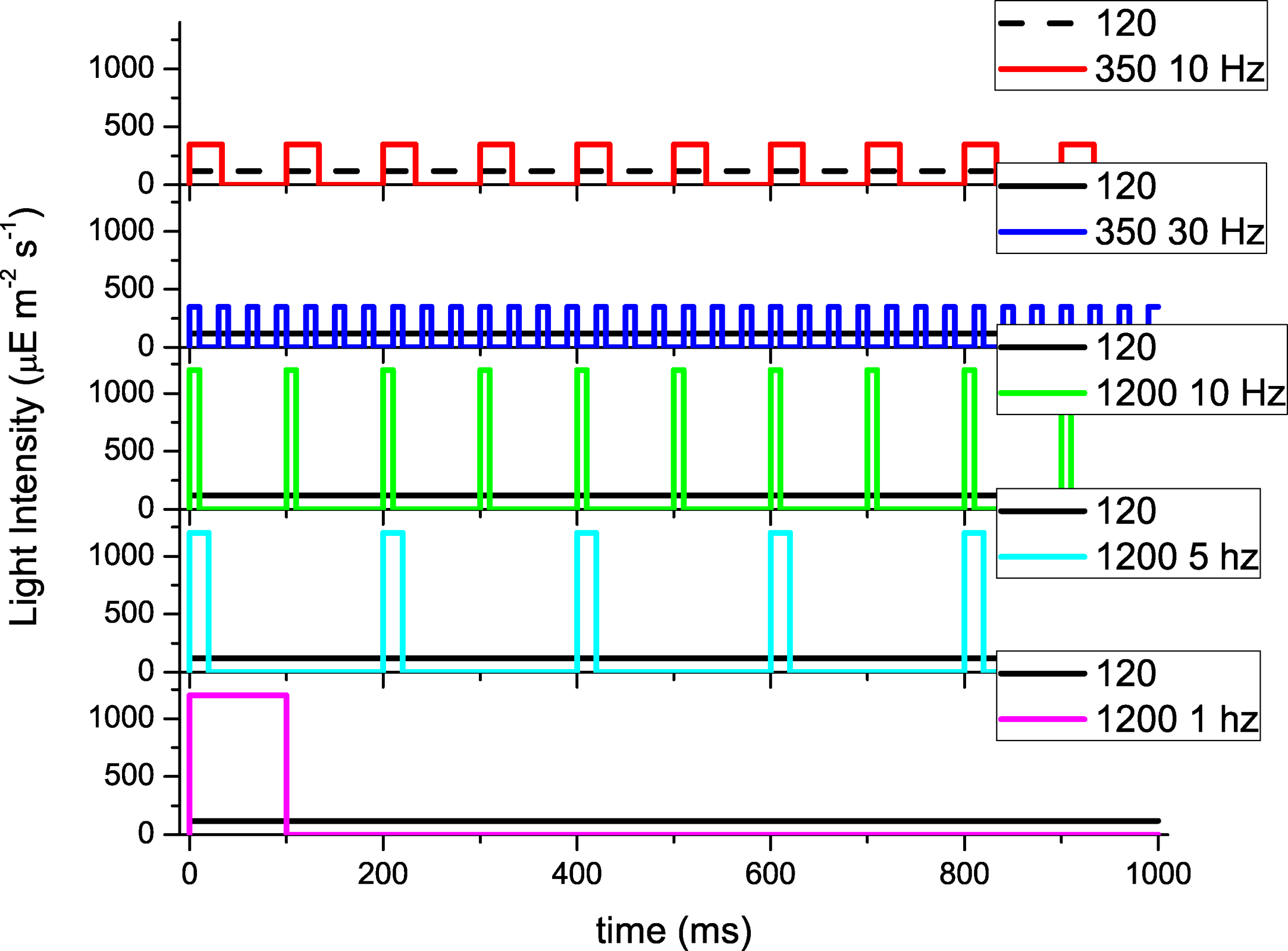

Supplement: Figure S2 — Pulsed light conditions utilized for Nannochloropsis salina growth. Alternated cycles of light and dark were designed to have all the same integrated light intensity (Ia), corresponding to 120 µE m−2 s−1 of continuous light. Flashes of two different intensities were employed, 350 and 1200 µE m−2 s−1, with a duty cycle of respectively 0.33 and 0.1. Light changes were performed with different frequencies, respectively 10 and 33 Hz with 350 µE m−2 s−1 and 10, 5 and 1 with 1200 µE m−2 s−1. These pulsed light conditions resulted in precise durations of flashes (t f) and dark (t d), as reported in Table 1. (TIF) [file pone.0038975.s002.tif]

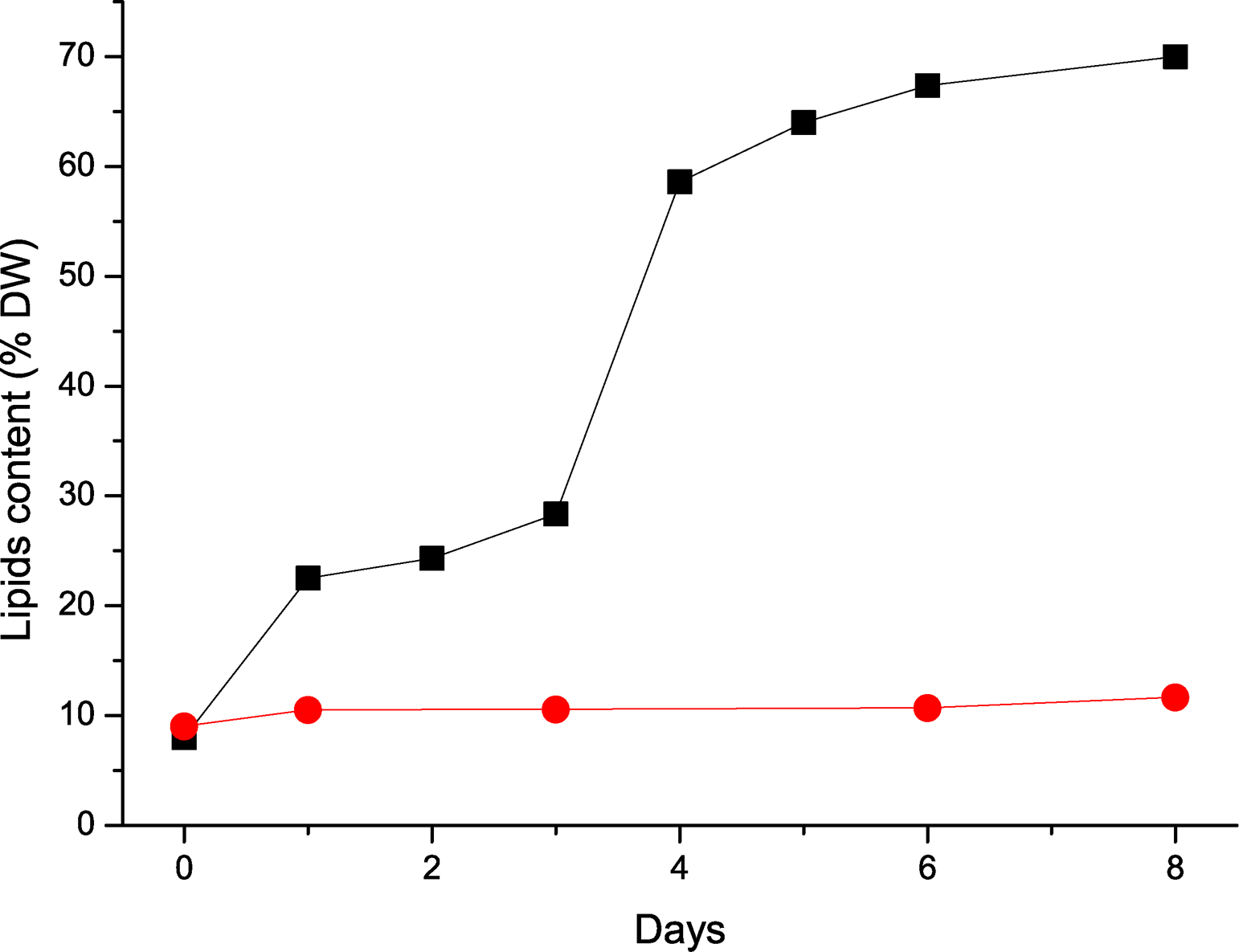

Supplement: Figure S3 — Timeline of lipids accumulation in Nannochloropsis cells exposed to 350 (black) and 150 (red) µE m −2 s −1 . Lipid content was evaluated each day using Nile Red staining correlated to total lipid concentration quantified gravimetrically (see Figure S4). (TIF) [file pone.0038975.s003.tif]

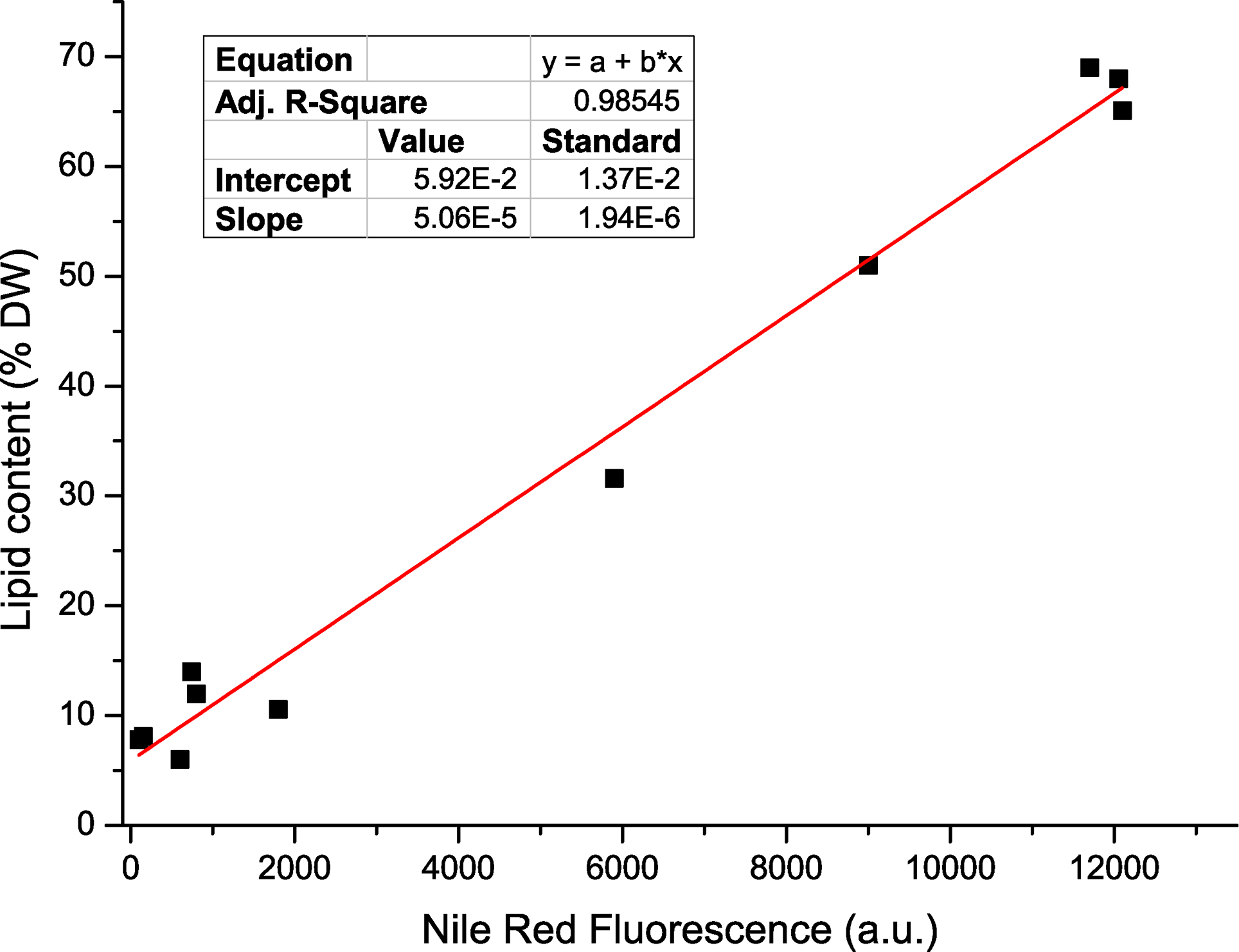

Supplement: Figure S4 — Correlation of lipid accumulation in Nannochloropsis evaluated by Nile Red staining and gravimetric analysis. (TIF) [file pone.0038975.s004.tif]
